# Correlation between serum SIRT1 and EZH2 expressions and peritoneal function in patients with diabetic nephropathy undergoing peritoneal dialysis

**DOI:** 10.12669/pjms.38.8.5527

**Published:** 2022

**Authors:** Qi Zhu, Haiyan Hu, Fang Xia

**Affiliations:** 1 Qi Zhu, Department of Trauma Surgery, Hanchuan people’s hospital, Hanchuan 431600, Hubei, P.R. China; 2 Haiyan Hu, Department of endocrinology, Affiliated Suizhou Hospital, Hubei University of Medicine, Suizhou 441300, Hubei, P.R. China; 3 Fang Xia, Department of Nephrology, Jingmen Hospital of Traditional Chinese Medicine, Jingmen 448000, Hubei, P.R. China

**Keywords:** Diabetic nephropathy, Silent information regulator 1, Zeste 2 polycomb repressive complex 2 subunit, Peritoneal function

## Abstract

**Objectives:**

To determine the correlation between the expression of silent information regulator 1 (SIRT1) and enhancer of zeste two polycomb repressive complex two subunit (EZH2) and peritoneal function in patients with diabetic nephropathy (DN) and peritoneal dialysis

**Methods:**

One hundred forty patients with DN underwent peritoneal dialysis in Hanchuan People’s Hospital from April 2016 to January 2019 were divided into four groups according to the duration of peritoneal dialysis. The levels of SIRT1 and EZH2 in serum were measured. The ratio of dialysate to plasma creatinine (D/Pcr) and the concentration of cancer antigen 125 (CA125) in peritoneal dialysate were determined. The ratio of urea clearance to urea distribution volume (Kt/V) of dialyzer was calculated. The correlations between SIRT1 and EZH2 expressions and peritoneal function were analyzed.

**Results:**

With the prolongation of peritoneal dialysis, serum SIRT1 expression, Kt/V, Ccr and CA125 decreased, while EZH2 expression and D/Pcr increased in patients with DN undergoing peritoneal dialysis. Pearson’s correlation analysis showed that SIRT1 expression was negatively correlated with D/Pcr while positively correlated with Kt/V and CA125, and serum EZH2 expression was negatively correlated with Kt/V and CA125 while positively correlated with D/Pcr. ROC analysis demonstrated that SIRT1 and EZH2 expressions had certain diagnostic value for the poor prognosis of patients with DN undergoing peritoneal dialysis.

**Conclusion:**

Serum SIRT1 and EZH2 expressions in patients with DN undergoing peritoneal dialysis are closely related to their peritoneal function. They have certain diagnostic value for the poor prognosis of patients with DN undergoing peritoneal dialysis.

## INTRODUCTION

Diabetic nephropathy (DN) is one of the common complications of diabetic patients, and one of the main causes of end-stage renal disease (ESRD), with unclear pathogenesis, which is still a challenge of clinical treatment.^1^,^2^ At present, this disease is mainly treated with renal replacement therapy.^3^ Among them, peritoneal dialysis can regularly renew the dialysate, which can effectively remove the toxic substances in the body, maintain the residual renal function, supplement the essential substances for the body, and improve the quality of life of patients to a certain extent.^4^ Peritoneal function is of great significance to the life of patients and success of surgery. Therefore, it is of great value to evaluate the peritoneal function of patients with DN undergoing peritoneal dialysis. Silent information regulator 1 (SIRT1) is a histone deacetylase, which is expressed in tissues and organs such as the liver and kidney.^5^ SIRT1 plays a key role in the occurrence and development of DN, and can improve the metabolism and enhance the ability to resist external stress of the body.^6^

Low SIRT1 expression can lead to the dysfunction of renal cells such as podocytes and glomerular mesangial cells.^7^,^8^ Enhancer of zeste polycomb repressive complex 2 subunit (EZH2) is a histone methyltransferase, which has been found to trigger DN occurrence by promoting renal fibrosis, and be highly expressed in DN patients.^9^,^10^ However, the studies on the correlations between SIRT1 and EZH2 expression levels and peritoneal function in patients with DN undergoing peritoneal dialysis are few. Therefore, in this study, based on the abnormal expressions of SIRT1 and EZH2 in DN patients, the correlations of serum SIRT1 and EZH2 expressions with peritoneal function in patients with DN undergoing peritoneal dialysis were explored.

## METHODS

A total of 140 patients with DN who underwent peritoneal dialysis in Hanchuan People’s Hospital from April 2016 to January 2019 were selected as subjects, including 74 males and 66 females aging 43-78 years (average, 56.4 ± 12.5 years), with a course of disease of 1-5 years (average, 3.2 ± 0.6 years). According to the duration of peritoneal dialysis, they were divided into initial peritoneal dialysis group (duration ≤ six months, n= 31), short-term peritoneal dialysis group (six months < duration ≤ three years, n = 38), mid-term peritoneal dialysis group (three years < duration ≤ 5 years, n = 39) and long-term peritoneal dialysis group (duration > five years, n = 32). No statistically significant differences were found in age or gender among each group (*p>* 0.05).

### Ethical Approval:

The study was approved by the Institutional Ethics Committee of Hanchuan People’s Hospital at July 24, 2021(No.:2021006), and written informed consent was obtained from all participants.

### Inclusion criteri

- Type-2 diabetes mellitus diagnosed based on the Diagnostic Criteria for Diabetes
- All patients meeting the diagnostic criteria for ESRD^2^;
- No peritonitis three months before the experiment.

### Exclusion criteria:

- Complicated with malignant tumors or connective tissue disease;
- Treated with hormones or immune drugs within one year;
- Other tissue infections within three months.

Full-automatic biochemical analyzer was purchased from Bio-Rad, USA. Baxter gambor dialyzer was purchased from Baxter, USA. SIRT1 and EZH2 ELISA kits, CA125 ELISA kit and creatinine ELISA kit was purchased from Invitrogen, USA.

***Pre-treatment of blood samples***: Fasting peripheral blood (3-5 mL) was collected from patients with DN undergoing peritoneal dialysis. After standing at room temperature for 20 min., the samples were centrifugated at 3000 × g for 10 min at 4°C. The serum was collected, packed into a specific centrifuge tube and stored in a refrigerator at -80°C for subsequent determination of corresponding indicators.

The expression levels of SIRT1 and EZH2 in the serum were measured by enzyme-linked immunosorbent assay (ELISA) using SIRT1 and EZH2 ELISA kits in accordance with the manufacturer’s instructions.

The dialysate-to-plasma creatinine ratio (D/Pcr) was determined with standard peritoneal equilibration test. One day in advance, 2.5% glucose dialysate was infused into the abdominal cavity overnight. On the next day, the dialysate was released and 2.5% dialysate was infused into the abdominal cavity. Four hour later, the concentrations of creatinine in the dialysate and plasma were determined using corresponding ELISA kits, and the D/Pcr was calculated. The ratio of urea clearance to urea distribution volume (Kt/V) of dialyzer (calculated according to the urea clearance on dialyzer and the total content of urea in the human body) and creatinine clearance rate (Ccr) (after emptying urine at eight a.m., 24-hour urine was collected and added with 4mL toluene for antisepsis, and then the concentrations of creatinine in the urine and plasma were detected using corresponding ELISA kits, Ccr = urinary creatinine concentration × 24-h urine volume/plasma creatinine concentration) were recorded in a certain dialysis time. The concentration of cancer antigen 125 (CA125) in peritoneal dialysate was detected using a radioimmunoassay kit.

All the patients with DN undergoing peritoneal dialysis were followed up for one year, with the deadline of January 2020. According to the follow-up results, the patients were divided into survived group and dead group, without loss to the follow-up.

### Statistical Analysis:

Statistical analysis was carried out using SPSS 25.0. The measurement data were described as mean ± standard deviation (x̄±*S*), and compared between two groups with the *t*-test and among multiple groups by the analysis of variance. The enumeration data were described as n, and compared between groups using the χ^2^ test. The correlations between SIRT1 and EZH2 expressions and peritoneal function parameters in patients with DN undergoing peritoneal dialysis were analyzed using Pearson’s correlation analysis. The diagnostic value of serum SIRT1 and EZH2 expressions in the poor prognosis of patients with DN was analyzed by the receiver operating characteristic (ROC) curve. P<0.05 was considered as statistically significant.

## RESULTS

With the prolongation of peritoneal dialysis, serum SIRT1 expression, Kt/V, Ccr and CA125 presented decreasing trends successively (*p<* 0.05), while EZH2 expression and D/Pcr showed successive increasing trends (*p<* 0.05) in patients with DN undergoing peritoneal dialysis (Table-I).In patients with DN undergoing peritoneal dialysis, serum SIRT1 expression was negatively correlated with D/Pcr (r = -0.781, *p<* 0.05) while positively correlated with Kt/V and CA125 (r = 0.621, r = 0.691, *p<* 0.05), and serum EZH2 expression was negatively correlated with Kt/V, Ccr and CA125 (r = -0.682, r = -0.724, *p<* 0.05) while positively correlated with D/Pcr (r = 0.820, *p<* 0.05), as seen in Table-II.

**Table-I:** Serum SIRT1 and EZH2 expressions and peritoneal dialysis-related indicators in patients with DN undergoing peritoneal dialysis of different groups.

Group	n	SIRT1 (ng/mL)	EZH2 (pg/mL)	D/Pcr	Kt/V	Ccr (L/1.73m^2^)	CA125 (U/mL)
Initial peritoneal dialysis group	31	39.82 ± 7.06	103.1 ± 21.03	0.49 ± 0.08	2.11 ± 0.70	66.68 ± 8.33	17.93 ± 3.02
Short-term peritoneal dialysis group	38	34.31 ± 6.86^a^	148.5 ± 26.44^a^	0.58 ± 0.10^a^	1.91 ± 0.52^a^	65.13 ± 8.46	17.04 ± 3.04
Mid-term peritoneal dialysis group	39	22.49 ± 4.98^ab^	186.2 ± 32.78^ab^	0.64 ± 0.12^ab^	1.73 ± 0.41^ab^	63.54 ± 8.04	16.10 ± 2.02^a^
Long-term peritoneal dialysis group	32	16.83 ± 3.06^abc^	231.6 ± 41.51^abc^	0.71 ± 0.14^abc^	1.69 ± 0.38^ab^	61.78 ± 8.74^a^	15.32 ± 2.03^ab^
F	-	111.706	100.299	27.995	4.602	2.715	6.274
p	-	0.000	0.000	0.000	0.004	0.045	0.001

***Notes:*** Compared with the initial peritoneal dialysis group,

a p< 0.05; compared with the mid-term peritoneal dialysis group,

^b^ p< 0.05; compared with the long-term peritoneal dialysis group, ^c^p< 0.05.

**Table-II:** Correlations of serum SIRT1 and EZH2 expressions with transport parameters.

	SIRT1		EZH2	

	r	p	r	p
D/Pcr	-0.781	< 0.05	0.820	< 0.05
Kt/V	0.621	< 0.05	-0.682	< 0.05
Ccr	0.177	> 0.05	-0.132	> 0.05
CA125	0.691	< 0.05	-0.724	< 0.05

The patients with DN undergoing peritoneal dialysis were followed up, revealing 57 patients died and 83 survived, with the mortality of 40.71%. Serum SIRT1 expression was (36.39 ± 7.35) ng/mL and (19.38 ± 3.07) ng/mL, and EZH2 expression was (121.7 ± 24.32) ng/mL and (203.2 ± 47.51) ng/mL, respectively, in the survived group and dead group. Compared with the survived group, SIRT1 expression, D/Pcr and Kt/V in the serum of the dead group decreased significantly (*p<* 0.05), while EZH2 expression, Ccr and CA125 increased significantly (*p<* 0.05), as shown in Table-III.

**Table-III:** Comparison of serum SIRT1 and EZH2 expressions and peritoneal dialysis-related indicators between survived group and dead group.

Group	n	SIRT1 (ng/mL)	EZH2 (pg/mL)	D/Pcr	Kt/V	Ccr (L/1.73m2)	CA125 (U/mL)
Survived group	83	36.39 ± 7.35	121.7 ± 24.32	0.58 ± 0.12	1.64 ± 0.41	60.86 ± 7.01	12.36 ± 3.01
Dead group	57	19.38 ± 3.07	203.2 ± 47.51	0.86 ± 0.13	1.81 ± 0.50	63.39 ± 7.14	17.34 ± 4.13
t	-	20.765	8.598	13.11	2.202	2.082	8.253
p	-	0.000	0.000	0.000	0.029	0.039	0.000

**Fig.1:**
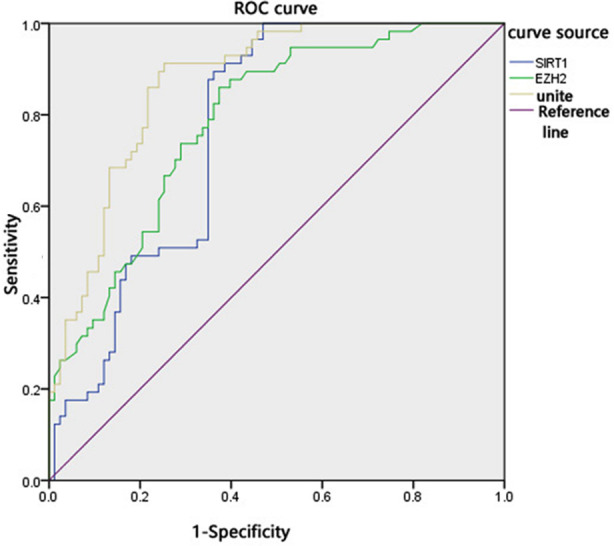
ROC map for diagnostic value of serum SIRT1 and EZH2 expressions in poor prognosis of patients with DN undergoing peritoneal dialysis.

ROC analysis results demonstrated that the area under the curve (AUC) of serum SIRT1 expression in diagnosing the poor prognosis of patients with DN undergoing peritoneal dialysis was 0.767 (95% CI: 0.689-0.844), with the sensitivity, specificity and cut-off value of 89.5%, 73.9% and 23.73 ng/ml, respectively. Additionally, the AUC of serum EZH2 expression in diagnosing the poor prognosis of patients with DN undergoing peritoneal dialysis was 0.787 (95% CI: 0.718-0.861), with the sensitivity, specificity and cut-off value of 86.2%, 72.7% and 135.27 ng/ml, respectively. The AUC of their combination was 0.871 (95% CI: 0.814-0.928), with the sensitivity and specificity of 86.0% and 78.3%. Compared with SIRT1, the diagnostic value of their combination in the poor prognosis of patients was increased (*p<* 0.05) Table-IV, Fig.1

**Table-IV:** Diagnostic value of serum SIRT1 and EZH2 expressions in poor prognosis.

Group	AUC	Cut-off value	Sensitivity (%)	Specificity (%)	95%CI
SIRT1	0.767	23.73 ng/mL	89.5	73.9	0.689-0.844
EZH2	0.787	135.27 pg/mL	86.2	72.7	0.712-0.861
Combination	0.871	-	86.0	78.3	0.814-0.928
Z_1_, p	2.104, 0.035				
Z_2_, p	1.757, 0.079				

## DISCUSSION

ESRD and chronic renal diseases are complications of diabetes, always accompanied by severe hypertension, renal hypertrophy and proteinuria.^12^ With the accelerating pace of modern life and the changes in dietary habits, the incidence of diabetes is increasing, thus leading to increasing incidence of DN and rapid DN progression, which often causes pathological changes in the heart, brain and eye.^13^ At present, peritoneal dialysis is commonly used to treat DN patients.^4^ Peritoneal failure is the main cause of surgical failure and poor prognosis. Moreover, reduced peritoneal function can cause severe hypertension, malnutrition and heart failure in patients with DN undergoing peritoneal dialysis, which is one of the main causes of death.^14^ Therefore, it is very important to timely evaluate the peritoneal transport function of patients with DN undergoing peritoneal dialysis for their health and quality of life.

SIRT1, belonging to the histone acetylase family, is expressed in cells such as renal tubular epithelial cells and podocytes, with multiple physiological functions including resisting apoptosis, regulating energy metabolism, reducing cell injury, anti-inflammation and maintaining normal organ functioning.^15^ In addition, it has certain diagnostic value for diabetic complications.^16^ The occurrence of DN is related to podocyte injury. Down-regulation of SIRT1 expression may result in the decrease of mitochondrial energy supply and podocyte injury, and thereby causing kidney injury.^17^ DN and tubulointerstitial fibrosis also presents a certain correlation, and imbalance of renal tubular epithelial-to-mesenchymal transition (EMT) will lead to renal failure.^18^ It has been found that EZH2 may promote renal fibrosis by affecting renal tubular EMT, thus participating in the occurrence and development of DN.^10^ Therefore, we speculate that SIRT1 and EZH2 may affect peritoneal function in patients with DN undergoing peritoneal dialysis by damaging podocytes and promoting renal fibrosis.

In this study, it was shown that with the prolongation of peritoneal dialysis, serum SIRT1 expression, Kt/V, Ccr and CA125 presented significant decreasing trends, while EZH2 expression and D/Pcr had significant increasing trends in patients with DN undergoing peritoneal dialysis. D/Pcr, Kt/V, Ccr and CA125 are closely related to peritoneal transport function, and Kt/V, D/Pcr and Ccr are relevant indicators to measure the adequacy of peritoneal dialysis, which can indirectly reflect the peritoneal transport function of patients undergoing peritoneal dialysis.^19^ CA125 is a glycoprotein antigen, which can reflect peritoneal transformation and the number of mesothelial cells, and is often used to measure the peritoneal transport function of patients with DN undergoing peritoneal dialysis.^20^

Pearson’s correlation analysis showed that SIRT1 expression was negatively correlated with D/Pcr while positively correlated with Kt/V and CA125, and serum EZH2 expression was negatively correlated with Kt/V and CA125 while positively correlated with D/Pcr, indicating that the expression levels of SIRT1 and EZH2 are closely related to the peritoneal function of patients. The worse the peritoneal transport function, the lower the expression level of SIRT1 and the higher the expression level of EZH2. Moreover, the patients with DN undergoing peritoneal dialysis were followed up, revealing 57 patients died, with the mortality of 40.71%. Compared with the survived group, SIRT1 expression, D/Pcr and Kt/V in the serum of the dead group decreased significantly, while EZH2 expression, Ccr and CA125 increased significantly, suggesting that the worse the peritoneal function, the higher the mortality. Early evaluation of peritoneal function and timely treatment may reduce the mortality in patients with DN undergoing peritoneal dialysis. Finally, ROC analysis demonstrated that the sensitivity and specificity of serum SIRT1 and EZH2 expressions in diagnosing the poor prognosis of patients with DN undergoing peritoneal dialysis were high. When SIRT1 expression level < 23.73 ng/mL and EZH2 expression level > 135.27 pg/mL, the patients with DN undergoing peritoneal dialysis were more likely to have poor prognosis, and treatment measures should be taken in time. The AUC of their combination in diagnosing the poor prognosis of patients with DN undergoing peritoneal dialysis was 0.871, which was higher than that of SIRT1 and EZH2 alone, but their combination presented no statistically significant difference with EZH2 in diagnostic value.

### Limitations of the study:

The number of subjects included in this study was limited, so the conclusions drawn may not be very convincing. In addition, this was a retrospective study with limited data integrity and homogeneity. It is necessary to further design a randomized controlled trial to verify the conclusions of this study.

## CONCLUSION

Serum SIRT1 and EZH2 expressions in patients with DN undergoing peritoneal dialysis are closely related to their peritoneal function. They have certain diagnostic value for the poor prognosis of patients with DN undergoing peritoneal dialysis. However, the mechanism of abnormal SIRT1 and EZH2 expressions in patients with DN undergoing peritoneal dialysis is still unclear, and further research is needed.

### Authors’ Contributions:

**QZ & FX:** Designed this stud, prepared this manuscript, are responsible and accountable for the accuracy and integrity of the work.

**HH:** Collected and analyzed clinical data, also significantly revised this manuscript.
